# Anatomy-driven multiple trajectory planning (ADMTP) of intracranial electrodes for epilepsy surgery

**DOI:** 10.1007/s11548-017-1628-z

**Published:** 2017-06-15

**Authors:** Rachel Sparks, Vejay Vakharia, Roman Rodionov, Sjoerd B. Vos, Beate Diehl, Tim Wehner, Anna Miserocchi, Andrew W. McEvoy, John S. Duncan, Sebastien Ourselin

**Affiliations:** 1 Centre for Medical Image Computing, University College London, London, UK; 2 Department of Clinical and Experimental Epilepsy, University College London Institute of Neurology, London, UK; 3 National Hospital for Neurology and Neurosurgery (NHNN), London, UK; 4 Dementia Research Centre, Department of Neurodegenerative Disease, University College London Institute of Neurology, London, UK

**Keywords:** Image-guided interventions, Neurosurgery, Computerized decision support, Trajectory planning, Epilepsy

## Abstract

**Purpose:**

Epilepsy is potentially curable with resective surgery if the epileptogenic zone (EZ) can be identified. If non-invasive imaging is unable to elucidate the EZ, intracranial electrodes may be implanted to identify the EZ as well as map cortical function. In current clinical practice, each electrode trajectory is determined by time-consuming manual inspection of preoperative imaging to find a path that avoids blood vessels while traversing appropriate deep and superficial regions of interest (ROIs). We present anatomy-driven multiple trajectory planning (ADMTP) to find safe trajectories from a list of user-defined ROIs within minutes rather than the hours required for manual planning.

**Methods:**

Electrode trajectories are automatically computed in three steps: (1) *Target Point Selection* to identify appropriate target points within each ROI; (2) *Trajectory Risk Scoring* to quantify the cumulative distance to critical structures (blood vessels) along each trajectory, defined as the skull entry point to target point. (3) *Implantation Plan Computation:* to determine a feasible combination of low-risk trajectories for all electrodes.

**Results:**

ADMTP was evaluated on 20 patients (190 electrodes). ADMTP lowered the quantitative risk score in 83% of electrodes. Qualitative results show ADMTP found suitable trajectories for 70% of electrodes; a similar portion of manual trajectories were considered suitable. Trajectory suitability for ADMTP was 95% if traversing sulci was not included in the safety criteria. ADMTP is computationally efficient, computing between 7 and 12 trajectories in 54.5 (17.3–191.9) s.

**Conclusions:**

ADMTP efficiently compute safe and surgically feasible electrode trajectories.

## Introduction

### Clinical background

One-third of patients with focal epilepsy have poor seizure control despite pharmaceutical treatment [11]. These patients are candidates for curative resective surgery if the epileptogenic zone (EZ) can be located and the EZ does not overlap with eloquent cortex. Non-invasive imaging can definitively identify the EZ in many patients; however, approximately 25% require invasive surgical intervention to identify the EZ [5]. One such intervention involves implanting stereo-electroencephalographic (SEEG) intracranial depth electrodes to target deep and superficial regions of interest (ROIs) within the cortex. Implanted electrodes may also map eloquent areas (e.g. motor or sensory cortex) and aid in determining safe resection margins [6].

**Fig. 1:**
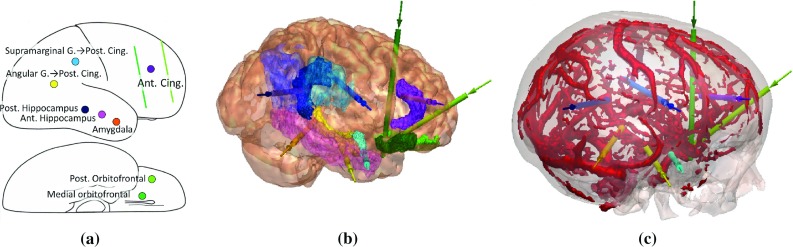
**a** A schematic strategy for a patient. Each *coloured circle* corresponds to a requested electrode with the required ROIs listed. When two ROIs are given, the first corresponds to the superficial ROI the electrode should traverse and the second corresponds to the deep ROI the electrode tip should be placed in. The corresponding implantation plan with **b** ROIs (*colours* corresponds to those given in the strategy) and **c** the skull (*white*) and blood vessels (*red*)

Implantation planning of SEEG electrodes is a two-stage process. In the first stage, a strategy consisting of a list of deep and superficial ROIs to record from is generated by a multi-disciplinary team of epileptologists, neurophysiologists, and neurosurgeons. Figure 1a displays an example strategy for a patient. In the second stage, neurosurgeons use the list of ROIs to guide precise planning of each electrode trajectory. Figure 1b displays the implantation plan with the ROI each electrode must attain. Figure 1c displays the implantation plan with blood vessels, important to consider when placing trajectories to minimize the risk of haemorrhage, and the skull, important to ensure the skull entry is surgically feasible. Finally, electrode trajectories are also placed to maximize their recording from grey matter (GM), as epileptic seizures arise in GM rather than white matter.

In current clinical practice, electrode trajectories are determined by manual placement and visual inspection of preoperative imaging data to ensure all surgical criteria are satisfied [13]. This is a complex, time-consuming task typically requiring between 2 and 3 h per patient. Automated trajectory planning algorithms may reduce planning time, improve safety, and increase GM sampling by optimizing trajectories according to quantitative measures of suitability.

### Previous work in automated trajectory planning

Automated single trajectory planning algorithms have been presented to maximize the distance to blood vessels and satisfy other surgical constraints for individual trajectories [2, 7, 8, 14, 17, 18, 20]. Each method defines a unique risk score based on specific surgical criteria, in general, trajectories near blood vessels or other critical structures are given high weights, and the lowest risk trajectory is selected. Most of these methods require the user to specify a target as a point [2, 8, 14, 17, 18, 20]. Essert et al. [7] requires the user to manually specify an ROI, as this method was applied to deep brain stimulation (DBS) the ROI was either the subthalamic nucleus or the ventral intermediate nucleus. This method also constrained the entry point search to a user-defined region of the skull, greatly reducing the potential number of trajectories. These methods are limited when applied to SEEG electrode implantation as (1) the most suitable target point for deep ROIs may not be obvious or may correspond to large brain structures, (2) multiple electrodes are implanted and must be placed to avoid conflicts between electrodes and to maximize GM sampling.

Automated multiple trajectory planning algorithms have been designed for implantation planning of SEEG electrodes. These methods allow users to specify multiple target and entry ROIs [4, 16, 19]. de Momi et al. [4] determined trajectories by randomly sampling from user-defined target and entry ROIs. Each ROI was defined as a user-selected point and distance threshold; hence, each target can be considered a spherical ROI. The best trajectory for each electrode was found in terms of entry angle with the skull, avoidance of blood vessels, avoidance of sulci, and ensuring a minimum distance between electrodes. Sparks et al. [16] required the user to specify each target as a point, and entry points were defined by the points on the skull surface below an angle threshold. The best trajectory for each electrode was then found in terms of avoidance of critical structures, maximum GM sampling, and ensuring a minimum distance between electrodes. As in the case of single trajectory planning algorithms, target points may not be obvious.

Zelmann et al. [19] overcame the need to define candidate target points. The hippocampus and amygdala were selected as ROIs and targets were computed by sampling a Gaussian distribution defined on the ROI distance map. Hence, targets near the ROI centreline were preferentially sampled. The best trajectory for each electrode was found in terms of entry angle with the skull, avoidance of critical structures, maximum GM sampling, and ensuring a minimum distance between electrodes. The major limitations of this work are that only hippocampus and amygdala were used as ROIs. Furthermore, the assumption that points near the ROI centreline are desirable may not hold for all ROIs.

### Novel contributions

In Sparks et al. [15], we presented anatomy-driven multiple trajectory planning (ADMTP) to compute electrode trajectories from a list of user-defined ROIs. This method is flexible enough to target any anatomically defined ROI. However, in the previous ADMTP implementation it was assumed that the centre of the ROI was the optimal target. In this manuscript, we have improved the ADMTP algorithm [15] in the following respects:

1. “Candidate target point selection” section leverages a user-specified spatial prior to preferentially sample targets either (a) near the ROI centre, to capture GM within the ROI or (b) near the medial surface of the ROI, to capture deep GM within the ROI.
2. “Candidate target point selection” section clusters target points if multiple electrodes are placed in the same ROI to ensure improved spatial coverage of the ROI and unique targets for each electrode.
3. “Trajectory risk scoring” section relaxes the hard constraints (maximum length and entry angle) if no suitable trajectories can be found within the given constraints.

Additionally, we have performed a thorough qualitative analysis comparing ADMTP and manual implantation plans according to safety, surgical feasibility, and spatial coverage of the suspected EZ for trajectories, target and entry points.

## Methodology

Anatomy-driven multiple trajectory planning (ADMTP) requires a (a) brain parcellation, (b) segmentation of critical structures (arteries, veins, sulci), (c) segmentation of the skull, and (d) list of user-specified ROIs. The section “Image acquisition” provides details of the protocols used to obtain the necessary images, while the “Brain parcellation and critical structure extraction” section details the algorithms used for extracting the necessary structures from the imaging data. For *N* user-specified ROIs, ADMTP finds an implantation plan $V( N ) = [ v_{1}, \ldots , v_{N} ]$, where $v_{n}$ is the trajectory for the *n*th electrode. For $v_{n}$, a unique set of *M* candidate target points $T_{n,i} : i \in \{1, \ldots , M\}$ are calculated as described in “Candidate target point selection” section. *P* entry points $E_{n,j} : j \in \{1, \ldots , P\}$ are determined for each $T_{n,i}$ and for each trajectory defined by $T_{n,i}$ and $E_{n,j}$ a risk score is computed as described in “Trajectory risk scoring” section. Finally, *V*(*N*) is computed to avoid electrode interference as described in “Implantation plan computation” section.

### Image acquisition

MR imaging is performed on a GE 3T MR750 scanner with a 32-channel head coil. A 3D T1-weighted MPRAGE scan is performed with a field-of-view (FOV) of $224 \times 256 \times 256$ mm ($\hbox {AP} \times \hbox {LR} \times \hbox {IS}$) with an acquisition matrix of $224 \times 256 \times 256$ for a reconstructed voxel size of 1 mm isotropic (TE/TR/TI $=$ 3.1 / 7.4 / 400 ms; flip angle $11^{\circ }$; parallel imaging acceleration factor 2). A post-gadolinium T1-weighted scan is performed with an FSPGR sequence with a FOV of $224 \times 256 \times 256$ mm and acquisition and reconstruction matrix of $224 \times 256 \times 256$ (TE/TR $=$ 3.1 / 7.4 ms; flip angle $11^{\circ }$). MR angiography (MRA) and venography (MRV) are performed using a 3D phase-contrast sequence with an FOV of $220 \times 220 \times 148.8$ mm with an acquisition matrix of $384 \times 256 \times 124$ for a reconstructed voxel size of $0.43 \times 0.43 \times 0.6$ mm (flip angle $8^{\circ }$; parallel imaging acceleration factor 2). To highlight the arteries, the MRA is scanned with a velocity encoding of 80 cm/s (TE/TR $=$ 4.0 / 9.3 ms). For sensitivity to the venous circulation, the MRV is scanned with a velocity encoding of 15 cm/s (TE/TR $=$ 4.8 / 26.4 ms), fat suppression, and a saturation band inferior to the FOV.

### Brain parcellation and critical structure extraction

**Fig. 2:**
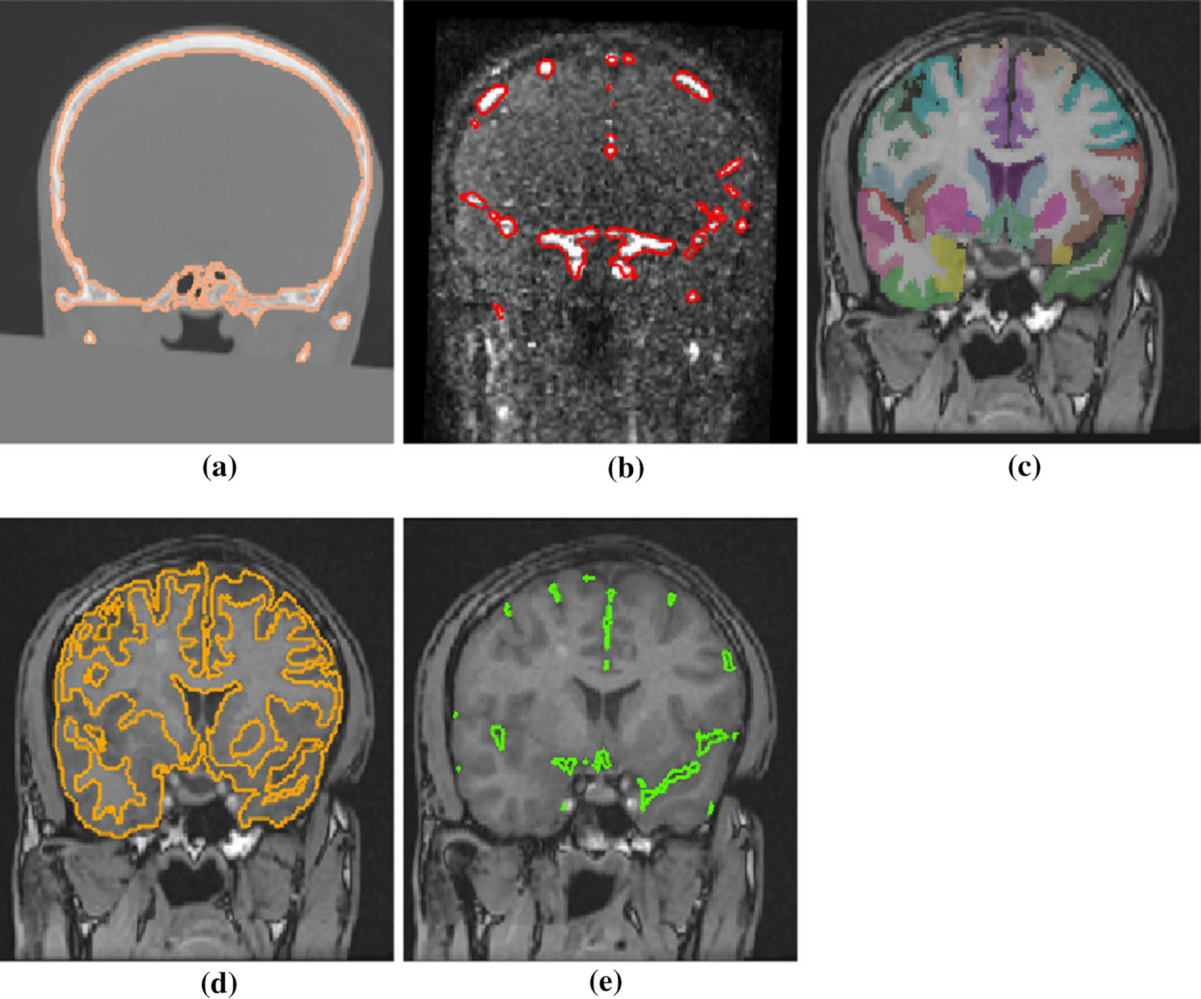
An example patient imaging dataset containing **a** CT with skull segmentation (*orange*), **b** MRV with vessel segmentation (*red*), **c** 3D T1-weighted MPRAGE with brain parcellation, **d** 3D T1-weighted MPRAGE with GM segmentation (*orange*), and **e** 3D T1-weighted MPRAGE with sulci segmentation (*green*)

The algorithms chosen for brain parcellation and segmentation of GM, arteries, veins, sulci, and skull are currently part of routine clinical practice for manual planning of SEEG electrode trajectories [12, 13]. Figure 2 shows examples of brain parcellation and structure segmentation. Veins and arteries are segmented from a post-gadolinium T1-weighted scan, MRV, and MRA using a multi-scale, multi-modal tensor voting algorithm [21]. The skull is segmented from CT using thresholding followed by morphologic operations for a connected surface.

Geodesic information flows (GIF) [3] is used to perform brain parcellation on a 3D T1-weighted MPRAGE using the Brain Collaborative Open Labeling Online Resource (BrainCOLOR) atlas [10] to define the anatomical labels. The brain atlas contains 142 possible regions. GM and sulci are extracted from the brain parcellation using thresholding.

### Candidate target point selection

Candidate target point selection is performed as follows. A risk map of the ROI is calculated as described in “Target risk map computation” section (as in [15]). Next, a novel algorithm is used to determine *M* unique target points $T_{n,i} : i \in \{1, \ldots , M\}$ for each electrode in the ROI, which are computed using *K*-means clustering of the candidate target points as described in “Candidate target point sampling” section.

#### Target risk map computation

A target risk map image is defined as $\mathcal {C}_{t} = [C, f_{t}(c): c \in C]$ where *C* is the grid of image voxel locations and *f*(*c*) is the target risk score for the given voxel *c*. *f*(*c*) is calculated as,

1 $$\begin{aligned} f(c) = {\left\{ \begin{array}{ll} 1, &{} \quad \text {if } c \notin \varOmega _\mathrm{roi} \\ 1, &{} \quad \text {if } c \in \varOmega _\mathrm{cri} \\ w_{sp} * f_\mathrm{sp}(c) + w_\mathrm{cri} * (1 - f_\mathrm{cri}(c)), &{} \quad \text {else} \end{array}\right. }\nonumber \\ \end{aligned}$$

$\varOmega _\mathrm{roi}$ and $\varOmega _\mathrm{cri}$ are the set of voxels in the ROI and critical structures (veins and arteries), respectively. Any pixel outside of the ROI or inside a critical structure is assigned the highest risk score and will not be considered as potential candidate target points. The weights $w_{sp}$ and $w_\mathrm{cri}$ control the relative importance of placing the target point using the spatial prior ($w_\mathrm{sp}$) or avoiding critical structures ($w_\mathrm{cri}$).

$f_\mathrm{cri}(c)$ and $f_\mathrm{sp}(c)$ are calculated as follows. The function $d(c, \phi )$ is the minimum distance between the point *c* and a structure $\phi$. Where $\phi$ is calculated using a bounding volume hierarchy (BVH) [20]. The distance is then normalized by $\tilde{d}(c, \phi ) = \frac{d(c, \phi )}{\max (d(b, \phi ))} : \forall b \in C$.

**Fig. 3:**
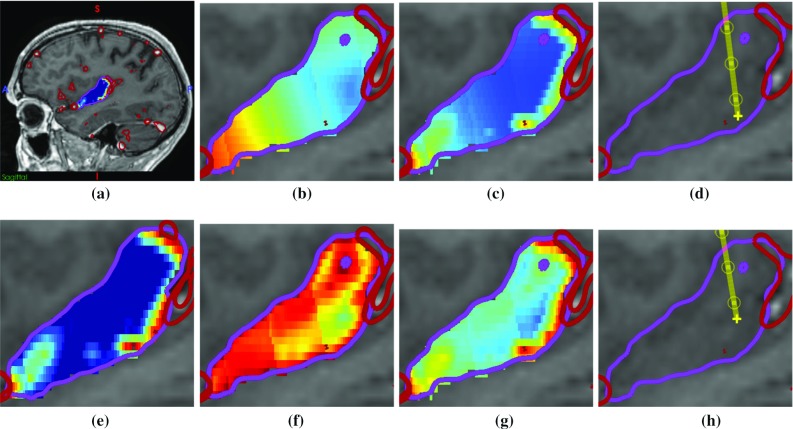
Example of the ROI centreline and medial surface spatial priors for the posterior insula. **a** Sagittal view of posterior insula (*purple*) with blood vessels (*red*) and $f_\mathrm{cri}(c)$ (heat map where blue is low risk and red is high risk). **e** A closer view of $f_{cri}(c)$. **b** $f_{sp}(c)$ for the ROI medial surface prior the inferior posterior area of the ROI is preferred (*blue*), **c** corresponding *f*(*c*) and **d** trajectory (*yellow*). **f** $f_{sp}(c)$ for the ROI centreline prior the thickest portion of the ROI is preferred (*blue*), **g** corresponding *f*(*c*) and **h** trajectory (*yellow*)

$f_\mathrm{cri}(c)$ is defined as $\tilde{d}(c, \phi )$ where $\phi$ is the union of all critical structures. The spatial prior $f_{sp}(c)$ is $\tilde{d}(c, \phi )$ where $\phi$ is either the skull, to prefer points near the medial surface of the ROI; or $\phi$ is the ROI, to prefer points near the centre of the ROI. The choice of spatial prior is user-defined according to clinical criteria, where using medial surface of the ROI prior provides targets in deep GM while the ROI centreline prior provides targets in GM of the ROI. Figure 3 provides an example of both spatial priors.

#### Candidate target point sampling

The set of pixels *Q* corresponding to local minima of *f*(*c*) is found using iterative flooding [1]. $p \in Q$ are then clustered into *K* groups using *K*-means clustering [9] where *K* is the number of electrodes in the ROI. For the *k*th electrode, the final target points are the *M* points in the *k*th cluster with the lowest values of *f*(*c*).

**Table 1:** The following values were set by a consensus of 3 neurosurgeons: the most oblique drillable angle, $d_\mathrm{ang}$, the minimum safe distance from blood vessels, $d_\mathrm{safe}$, the distance at which there is no risk, $d_\mathrm{risk}$, and the minimum distance between electrodes, $d_\mathrm{traj}$

Parameter	*M*	$w_\mathrm{roi}$	$w_\mathrm{cri}$	$d_\mathrm{len}$	$d_\mathrm{ang}$	$d_\mathrm{safe}$	$d_\mathrm{risk}$	$d_\mathrm{traj}$
Value	10	0.25	0.75	80 mm	$15^{\circ }$	3 mm	10 mm	10 mm

A commonly used electrode configuration determined the electrode length, $d_\mathrm{len}$. The following values were set empirically: the number of candidate targets, *M*, and the relative importance of the spatial prior, $w_\mathrm{roi}$, versus avoiding critical structures, $w_\mathrm{cri}$

### Trajectory risk scoring

A set of possible entry points defined as $E_{n,j} : j \in \{1, \ldots , P\}$ are computed by considering all vertices on the skull mesh. Potential trajectories $\overline{T_{n,i}E_{n,j}}$ are removed from consideration with the following criteria (modified from Zombori et al. [20]):

1. $\overline{T_{n,i}E_{n,j}}$ is longer than $d_\mathrm{len}$.
2. The angle between $\overline{T_{n,i}E_{n,j}}$ and the skull normal is greater than $d_\mathrm{ang}$.
3. $\overline{T_{n,i}E_{n,j}}$ does not traverse the superficial ROI, if specified.
4. $\overline{T_{n,i}E_{n,j}}$ intersects a critical structure (arteries, veins, or sulci).

If no suitable trajectories are found, the $d_\mathrm{ang}$ and $d_\mathrm{len}$ are iteratively relaxed such that $d_\mathrm{len}$ is increased by 10 mm (up to 110 mm) and $d_\mathrm{ang}$ is increased by $10^{\circ }$ (up to $45^{\circ }$) until a set of suitable trajectories is found.

For suitable trajectories, a weighted score $S_{n,i,j}$ representing a combination of a risk score $R_{n,i,j}$ and GM ratio $G_{n,i,j}$ is calculated. $R_{n,i,j}$, a measure of cumulative distance to blood vessels, is computed as:

2 $$\begin{aligned} R_{n,i,j} = \frac{\displaystyle \int _{E_{n,j}}^{T_{n,i}} d_\mathrm{risk} - (f_\mathrm{cri}(x)-d_\mathrm{safe} ) \hbox {d}x}{(d_\mathrm{risk}-d_\mathrm{safe}) * \hbox {length}}, \end{aligned}$$

where trajectories with $f_\mathrm{cri}(x)$ closer than $d_\mathrm{safe}$ have the highest risk ($R_{n,i,j}=1$) while $f_\mathrm{cri}(x)$ farther than $d_\mathrm{risk}$ have no risk ($R_{n,i,j}=0$).

$G_{n,i,j}$ is a measure of the proportion of electrode contacts, the parts of the electrode that record EEG signals, in GM. The electrode contact locations were modelled by selecting a commonly implanted electrode. Each electrode has $Q=10$ contacts placed at the points $p_q$ spaced in intervals of 10 mm along the trajectory starting at the target point $T_{n,i}$. Each contact is assessed on location in the GM at three points, the centre of the contact, $p_{q}$, and both ends of the contact, $p_q \pm p_r$, where $p_{r} = 1.2$ mm is the radius of the contact. Hence, each electrode contact may have a value of $\big [0, 1/3, 2/3, 1\big ]$. $G_{n,i,j}$ is the summation of GM capture over all contacts calculated as,

3 $$\begin{aligned} G_{n,i,j} = \frac{\displaystyle \sum \nolimits _{q = 1}^{Q} (H[f_\mathrm{gm}(p_q - p_r) ] + H[f_\mathrm{gm}(p_q) ] +H[ f_{gm}(p_q + p_r) ] }{3 * Q},\nonumber \\ \end{aligned}$$

where $f_\mathrm{gm}(\cdot )$ is the signed distance from the GM surface and $H[\cdot ]$ is the Heaviside function, with values of 1 inside GM and 0 outside.

The weighted score is computed as $S_{n,i,j} = 10 * R_{n,i,j} + G_{n,i,j}$. 10 was set empirically to prioritize low risk over a high GM ratio.

### Implantation plan computation

The final implantation plan *V*(*N*) is found by optimizing,

4 $$\begin{aligned}&S_\mathrm{total} = \mathop {\text {arg}\,\text {min}}\limits _{V(N)} {\left( \frac{1}{N} \sum _{n=1}^{N} S_{n,i,j} \right) } \nonumber \\&\quad \text { s.t. } D(\overline{T_{n,i} E_{n,j}}, \overline{T_{k,i} E_{k,j}} ) > d_\mathrm{traj} : \forall n, \forall k \in \{ 1, \ldots , N\}, n \ne k.\nonumber \\ \end{aligned}$$

where $d_\mathrm{traj}$ specifies the minimum distance between trajectories to ensure no conflicts. *V*(*N*) is computed using the depth-first graph search method described in Sparks et al. [16].

One modification has been made to the graph search strategy to improve performance. For an electrode conflict $D(\overline{T_{n,i} E_{n,j}}, \overline{T_{k,i} E_{k,j}} ) < d_\mathrm{traj}$ let the next considered trajectory be defined as $\overline{T_{k',i} E_{k',j}}$. In Sparks et al. [16], $\overline{T_{k',i} E_{k',j}}$ was the next lowest risk trajectory (i. e. $k+1$). To decrease computation time in this work, we calculate $\overline{T_{k',i} E_{k',j}}$ as the next lowest risk trajectory that satisfies the constraint $D(\overline{T_{k,i} E_{k,j}}, \overline{T_{k',i} E_{k',j}}) > 0.5 \text { mm}$. This allows for many spatial similar trajectories to be discarded decreasing the number of trajectory combinations considered. If no combination of trajectories exists which satisfies $d_\mathrm{traj}$, ADMTP returns the plan with the largest possible distance between trajectories.

## Experimental design and results

### Experimental design

ADMTP was evaluated on retrospective data from 20 patients with refractory focal epilepsy who underwent intracranial electrode implantation. Each patient had between 7 and 12 electrodes placed (190 total). Prior to trajectory planning a list of regions to target is determined by a multi-disciplinary team discussion consisting of expert epileptologists, neurophysiologists and neurosurgeons based on the evaluation of the clinical history, seizure semiology, ictal and interictal scalp EEG and results of multi-modal imaging. Following this manual plans (MPs) were determined by the consensus of two neurosurgeons, and some regions were removed from consideration if no safe trajectory to the target could be determined. The list of regions where a MP trajectory was determined were used to evaluate ADMTP with the parameters given in Table 1. ADMTP and MP trajectories corresponding to the same region were compared.

### Quantitative trajectory suitability

**Fig. 4:**
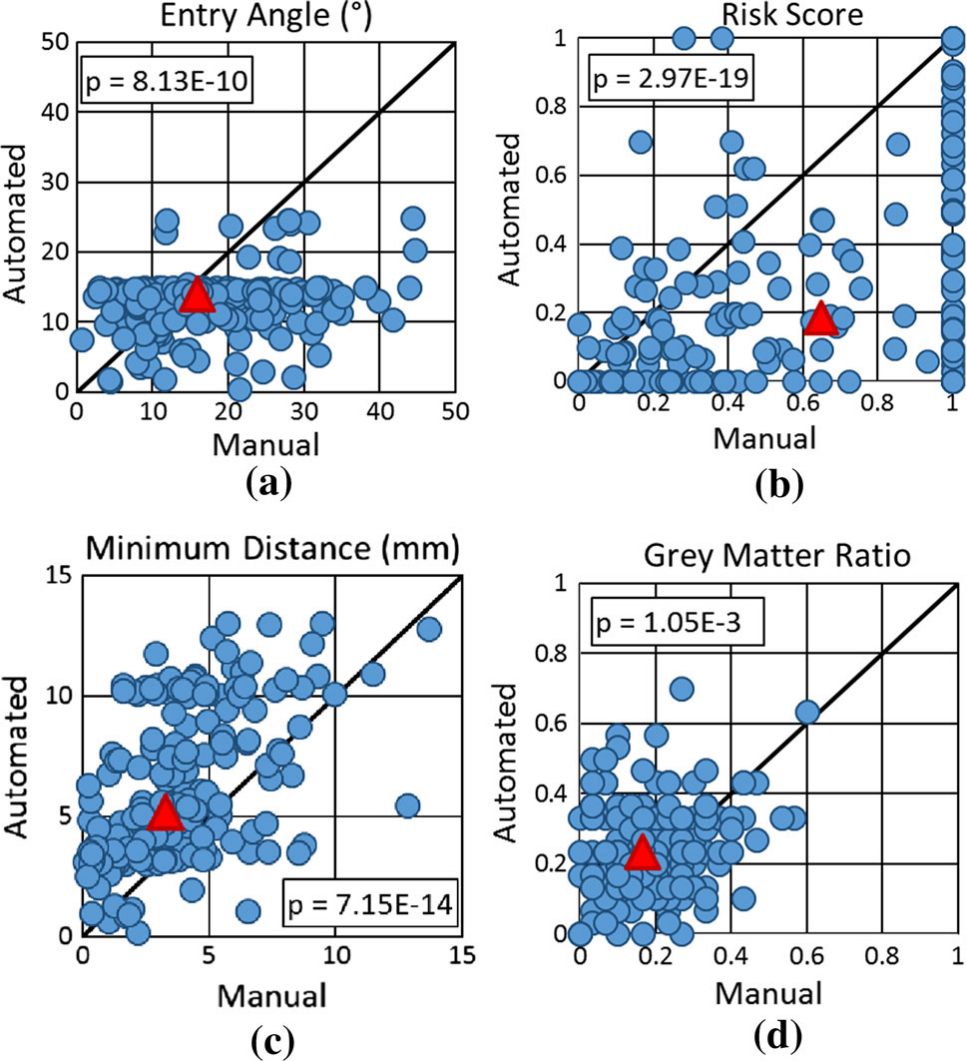
Measures of suitability for manual (plotted on the *X* axis) versus ADMTP (plotted on the *Y* axis) trajectory for **a** angle with respect to the skull surface normal, **b** risk score ($R_{n,i,j}$), **c** distance to the nearest critical structure, and **d** GM ratio ($G_{n,i,j}$). *Red triangles* are the centre of mass for each measure. For **a** angle and **b** risk score points below the diagonal correspond to ADMTP giving the preferred result. For **c** distance to critical structures and **d** GM ratio points above the diagonal correspond to ADMTP giving the preferred result

All 190 trajectories were assessed on the following quantitative measures of trajectory suitability: angle with respect to the skull surface normal, risk score ($R_{n,i,j}$), distance to nearest critical structure, and GM ratio ($G_{n,i,j}$). In Fig. 4, each point corresponds to one electrode with the manual trajectory value plotted on the *X* axis and the ADMTP trajectory value plotted on the *Y* axis. The red point represents the centre of mass for each measure. Points below the diagonal have a lower value for ADMTP compared to manual trajectories. For angle and risk score, points below the diagonal correspond to ADMTP giving the preferred value. For GM ratio and minimum distance points above the diagonal represent ADMTP giving the preferred value. A two-tailed Student’s *t* test evaluated the statistical significance between values determined by ADMTP and manual trajectories where the null hypothesis was that the methods return similar values.

ADMTP had a more feasible entry angle in 129 / 190 trajectories and increased GM sampling in 108 / 190 trajectories. ADMTP found trajectories that were safer in terms of reduced risk score (159 / 190 trajectories) and increased distance to the closest critical structure (155 / 190 trajectories). All differences between ADMTP and manual trajectories were statistically significant. The discrepancies between risk score and closest critical structure in 4 trajectories are due to risk being a cumulative distance measure; hence, it is maybe higher due to other critical structures that are not the closest critical structure.

**Fig. 5:**
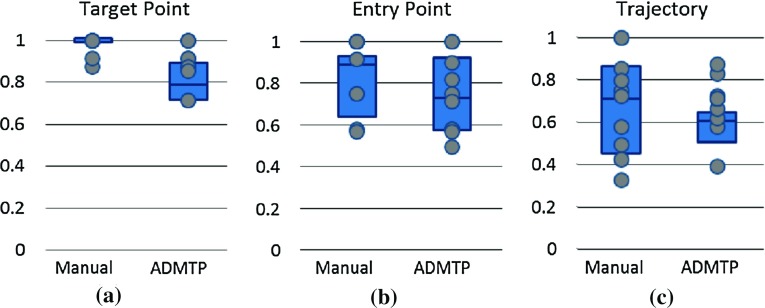
Fraction of trajectories per plan considered to have suitable **a** sampling of target ROIs, **b** surgically feasible entry points, and **c** safe trajectories. *Each grey dot* represents a plan with the *box plot* representing each quartile

### Qualitative implantation plan suitability

A neurosurgeon who did not participate in creating MPs and was blinded to plan origin assessed 10 randomly selected MPs and corresponding ADMTPs (94 electrodes). Implantation plans as a whole were assessed for suitable spatial coverage of the suspected EZ. Each trajectory was assessed according to whether they would proceed to implantation based on the following criteria.

1. *Target Point* is (a) sufficiently sampling appropriate GM and (b) safe.
2. *Entry Point* is (a) achievable surgically (i.e. drillable) and (b) safe.
3. *Trajectory* is safe.

In all cases, safety was assessed by being at least 3 mm away from all visible blood vessels and not crossing sulcal boundaries. In this study, crossing sulcal boundaries was included in safety criteria as small vessels within deep sulci may not be fully resolved with the current imaging protocol.

All implantation plans had suitable spatial coverage of the EZ. Figure 5 shows the results of the qualitative assessment per plan. MPs attained the appropriate target in 97% of cases, while ADMTP attained the appropriate target in 90% of cases. Table 2 details the reasons why target points were considered unsuitable, primarily due to placement in the wrong portion of the ROI. Table 3 lists the reasons why entry points and trajectories were considered infeasible. In both MPs and ADMTP plans, 30% of trajectories were considered too close to either blood vessels or sulci. If proximity to sulci was not included in the safety assessment, 85% of MP and 95% of ADMTP trajectories were considered suitable.

### Computational efficiency

**Fig. 6:**
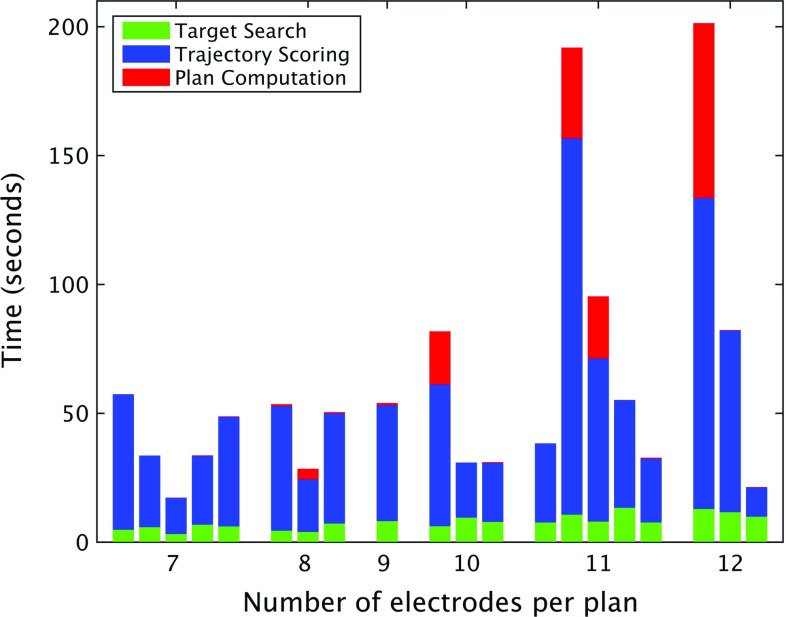
Computational time for each step in ADMTP: candidate target point search, entry point search and trajectory risk computation, and implantation plan computation for the number of electrodes in the plan

The computational efficiency of ADMTP was assessed for target point selection, trajectory risk scoring, and implantation plan computation. Computations were performed on a computer with a Intel(R) Xeon(R) 12 core CPU 2.10 GHz with 64.0 GB RAM and a single NVIDIA Quadro K4000 4GB GPU. Figure 6 reports computation time for each step in the process.

Automated planning took 54.5 s (17.3–191.9 s). For the majority of plans, most of the computation time was spent on trajectory risk scoring (11–146 s). As described in Zombori et al. [20] risk score computation time is dependent on the number of trajectories being considered. For target point search computation time is relatively linear with respect to the number of electrodes. Finally, for cases with a fewer number of electrodes (<10) plan computation time was insignificant (under 1 s), but this computation time increases substantially for more complex plans with a larger number of electrodes.

**Table 2:** Reasons for target point unsuitability

Reason	MP	ADMTP
Posterior	0	3
Shallow	1	5
Not in GM	1	1
Near sinus	0	1

**Table 3:** Reasons for entry point and trajectory unsuitability

Reason	Entry		Trajectory	
	MP	ADMTP	MP	ADMTP
Near vessel	13	21	14	5
Near sulci	1	4	15	24
Skull entry	0	1	–	–

## Discussion

A single neurosurgeon blinded to plan origin rated 30% of trajectories, from both MP and ADMTP, as being unsuitable. The number of infeasible trajectories is higher than those reported in our previous study [16] due to a stricter and better defined criteria for assessing trajectories as described in “Qualitative implantation plan suitability” section. Also, in the current study, traversing sulci was grounds for finding a trajectory unsuitable. If this criterion was not included, the success rate was 85% for MPs and 95% for ADMTP. In many epilepsy surgery centres, traversing sulci is not regarded as a major issue, provided large vessels have been identified and avoided.

Additionally, a different neurosurgeon evaluated plan suitability in this study compared to Sparks et al. [16], which may account for differences in the qualitative results. Our findings highlight that trajectory planning criteria for SEEG electrode implantation are non-obvious and may vary between neurosurgeons. Good automated trajectory planning may reduce variability by providing quantitative measures of trajectory suitability. In this current work, ADMTP performs similarly to neurosurgeons in terms of trajectory suitability. However, a larger qualitative study involving multiple neurosurgeons is required to better assess the suitability of ADMTP for trajectory planning.

**Fig. 7:**
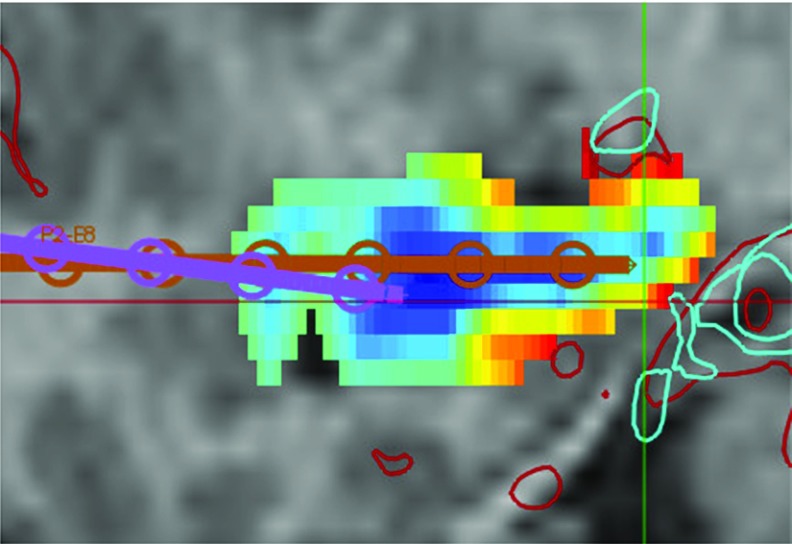
Electrode where ADMTP (*purple*) places the target shallow compared to the manual (*brown*) target. The presence of deep blood vessels results in a target risk score (*red* corresponds to high values and blue to low values) where points away from the ROI medial surface are preferred

Our qualitative results also highlight the need for good critical structure segmentation, especially of the small blood vessels and sulci, which are essential for computing surgically feasible plans. The vast majority of infeasible trajectories for ADMTP were caused by errors in the segmentation of critical structures resulting in inaccurate quantitative risk scores for some trajectories. In this work, MRA and MRV are used to segment blood vessels. A limitation of our current protocol is that small vessels located deep in the brain may not be segmented. As ADMTP is agnostic to the origin of critical structures, additional imaging protocols may be added to segment blood vessels which may be missed with the current protocol. An area of active research we are undertaking is determining the optimal protocol for detecting blood vessels to ensure safe, avascular trajectories.

**Fig. 8:**
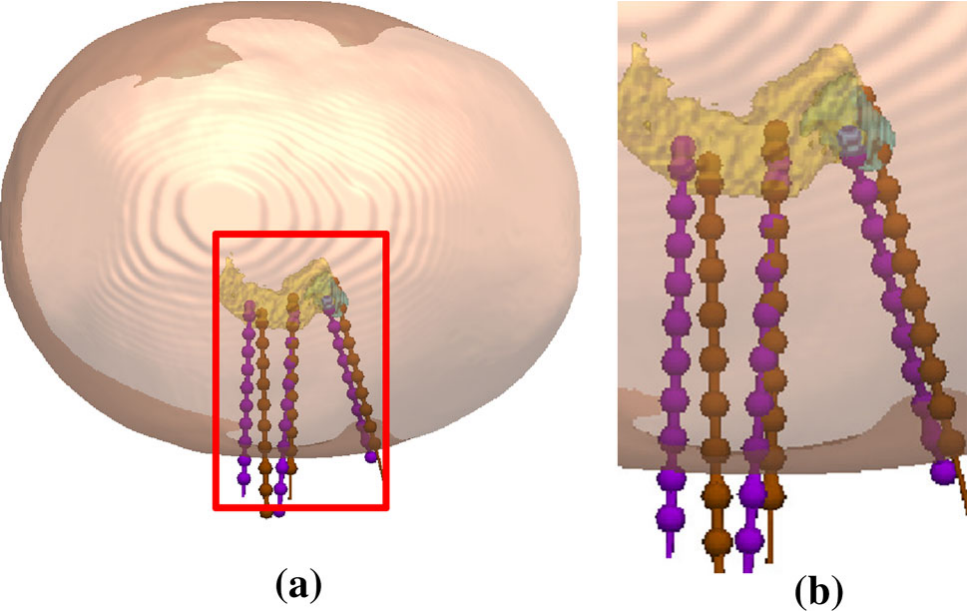
Electrode where ADMTP (*purple*) places the target posterior in the hippocampus (*yellow*) compared to the manual (*brown*) target. The two other electrodes displayed correctly target the amygdala (*cyan*) and the body of the hippocampus. **b** Corresponds to the *red box* in **a**

**Fig. 9:**
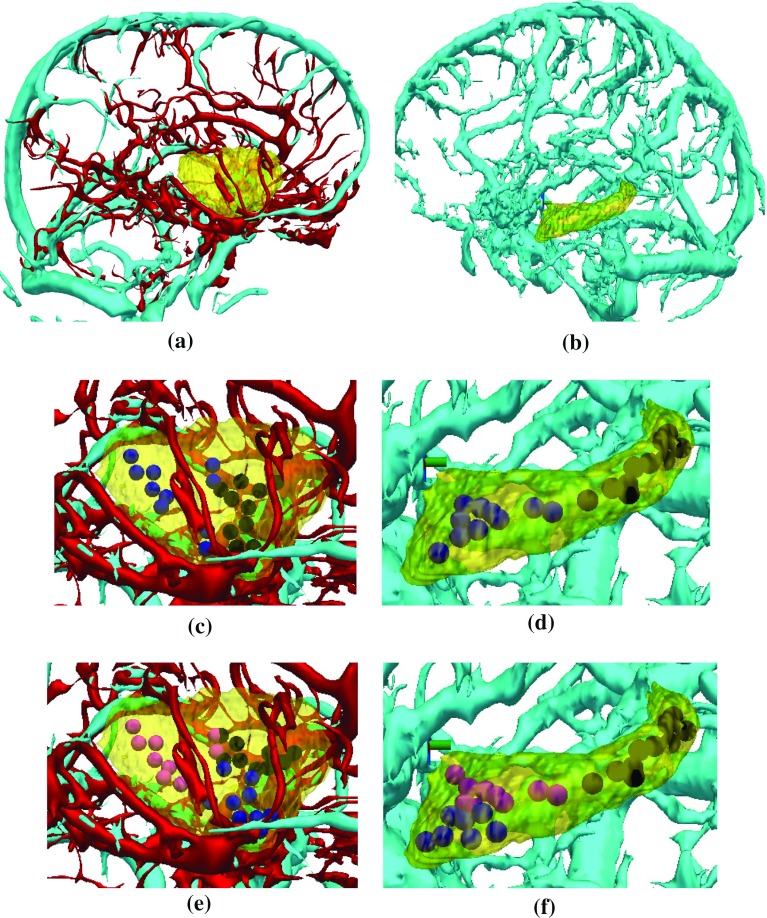
Example of target point clustering for **a** the anterior insula (*yellow*) and **b** hippocampus (*yellow*) with arteries (*red*) and veins (*cya*n). For the anterior insula **c** $K=2$ clusters target points along the anterior–posterior direction, while **e** $K=3$ clusters target points along anterior–posterior and inferior–superior directions. For the hippocampus target points clusters along the anterior-posterior direction for **d** $K=2$ and **f** $K=3$

ADMTP leverages two distinct spatial priors to place target points either near the ROI centreline or medial surface. The priors result in ADMTP placing target points in ROIs according to user-defined clinical criteria, ether internally to or traversing the ROI. The clinical criteria for which spatial prior to use is as follows:

1. Choose the ROI medial surface for targets on the cerebral cortex where the goal is to record from GM near deep sulci (e.g. leg region of the motor cortex).
2. Choose the ROI medial surface for deep targets where the goal is to record from GM near the medial surface of the brain (e.g. cingulate gyrus).
3. Choose the ROI centreline for targets where recording from GM within the ROI is important (e.g. hippocampus) or where there are vascular structures that are adjacent or near the medial surface of the ROI.

Learning spatial priors for specific ROI, such as the insula, using either historic implantation cases or clinically defined criteria is one area of future research.

The presence of midline cerebral blood vessels resulted in 5 target points being placed too shallow in the ROI. Figure 7 shows an example where the trajectory for ADMTP (purple) was placed shallow compared to the MP (brown) due to the presence of blood vessels (cyan, red). Three medial temporal lobe electrodes were placed too posterior in the ROI. In these cases, one electrode is placed in the amygdala correctly and one electrode is correctly placed in the body of the hippocampus. However, the other electrode is placed in the tail of the hippocampus to ensure no electrode conflicts (i.e. achieve an inter-electrode distance $>d_\mathrm{traj}$). Figure 8 shows an example with amygdala (cyan) and hippocampus (yellow) and the corresponding trajectories for ADMTP (purple) and MP (brown). In these cases, a more refined spatial prior may improve target point selection.

The *K*-means clustering used to identify separate candidate target points for each electrode returns unique clusters. Figure 9 shows examples of the clustering for $K=2$ and $K=3$ for the anterior insula and hippocampus. As *K*-means clustering is unsupervised, clusters are typically divided by the geometry of the ROI; however, these clusters may not represent areas desired by clinicians. With no changes to the ADMTP algorithm, a brain atlas containing different ROIs related to clearly defined targets (such as the anterior and posterior hippocampus) could provide better trajectory placement. This strategy would ensure no electrodes are placed within inappropriate areas of the ROI by, for instance, removing the posterior portion of the hippocampus that clinicians do not want to target.

## Concluding remarks

We presented an anatomy-driven multiple trajectory planning (ADMTP) algorithm to determine a set of intracranial electrode trajectories from user-defined anatomical regions of interest (ROIs). Compared to manual planning, ADMTP lowered risk in 83% of trajectories and increased GM sampling in 57% of trajectories. A single neurosurgeon blinded to plan origin found ADMTP returned suitable trajectories for 70% of electrodes. This is comparable to the 70% of trajectories considered suitable from manual plans. Trajectory acceptability was 85% (manual) and 95% (ADMTP) if traversing sulci was not included in the safety criteria. ADMTP efficiently calculates (<5 min) safe and surgically feasible trajectories for stereo-electroencephalography (SEEG) electrodes.
